# Characterization of Nanocomposites by Thermal Analysis 

**DOI:** 10.3390/ma5122960

**Published:** 2012-12-19

**Authors:** Carola Esposito Corcione, Mariaenrica Frigione

**Affiliations:** Department of Engineering for Innovation, University of Salento, Lecce 73100, Italy; E-Mail: mariaenrica.frigione@unisalento.it

**Keywords:** nanocomposites, thermal analysis, DSC, TGA, TMA, DTA

## Abstract

In materials research, the development of polymer nanocomposites (PN) is rapidly emerging as a multidisciplinary research field with results that could broaden the applications of polymers to many different industries. PN are polymer matrices (thermoplastics, thermosets or elastomers) that have been reinforced with small quantities of nano-sized particles, preferably characterized by high aspect ratios, such as layered silicates and carbon nanotubes. Thermal analysis (TA) is a useful tool to investigate a wide variety of properties of polymers and it can be also applied to PN in order to gain further insight into their structure. This review illustrates the versatile applications of TA methods in the emerging field of polymer nanomaterial research, presenting some examples of applications of differential scanning calorimetry (DSC), thermogravimetric analysis (TGA), dynamic mechanical thermal analysis (DMTA) and thermal mechanical analysis (TMA) for the characterization of nanocomposite materials.

## 1. Introduction

The combination of two different materials, for instance polymeric, is a simple route for combining the attractive features of different materials in order to enhance the deficient characteristics of a particular material [1]. Many common examples of composite materials can be found in the world around us. Wood and bone are examples of natural composites [2]. Recent and successful examples of improved properties that can be achieved by using these procedures are offered by adding to a polymeric phase of organic and inorganic filler, for instance hyperbranched polymers [3,4] and inorganic nanofillers.

In particular, polymer composites reinforced with inorganic fillers of dimensions in the nanometer range, known as nanocomposites, have attracted great interest from researchers, due to unexpected synergistic properties derived from the two components. The most studied polymer nanocomposites (PN) are composed of thermoplastic or thermosetting matrix, organically modified montmorillonite (OMMT) [5,6,7,8,9] and modified boehmite [10,11,12,13] or carbon nanotubes (CNTs) [14,15,16,17,18].

Polymer/clay nanocomposites are characterized by improved thermal, mechanical, barrier, fire retardant, and optical properties compared to either the matrix or to conventional composites, commonly called “particulate microcomposites”, because of their unique phase morphology derived by layer intercalation or exfoliation, that maximizes interfacial contact between the organic and inorganic phases and enhances bulk properties [19,20,21].

Since the discovery of carbon nanotubes (CNTs) in 1991 by Iijima [22], CNTs have been extensively studied by researchers in various fields such as chemistry, physics, materials science, and electrical engineering. Carbon nanotubes possess high flexibility, low mass density, and large aspect ratios (typically ca. 300–1000). They have a unique combination of mechanical, electrical, and thermal properties that make them excellent candidates to substitute or complement the conventional nanofillers in the fabrication of multifunctional polymer nanocomposites [14].

The rich bibliography on polymer/clay nanocomposites shows that the effect of montmorillonite on thermal properties of a matrix is complex and many factors contribute to the enhancement of the glass transition, such as OMMT dispersion, the interfacial strength, type of polymer matrix, preparation method, possible catalytic effects induced by organomodifier and/or montmorillonite itself, *etc.* [23]. Also in the case of carbon nanotubes, many studies report the importance of studying the thermal properties of nanocomposites containing CNTS, particularly with TGA, since a significant enhancement in thermal stability of the polymeric matrices filled with the nanotubes, compared to unfilled ones, was observed [8].

In PN, the efficiency of intercalation of the polymer in the lamellar galleries is usually measured by means of x-ray diffraction (XRD), transmission electronic microscopy (TEM), rheology, NMR, and real-space interface observations [24,25,26]. Although wide angle XRD offers a convenient method to determine the interlayer spacing of the silicate layers in the intercalated nanocomposites, little can be said about the spatial distribution of the silicate layers or on structural non-homogeneities in nanocomposites. On the other hand, TEM is very time-intensive, and only gives qualitative information on the sample as a whole, due to the small investigable area [27]. Differential scanning calorimeter (DSC) has been widely applied to the study of many phenomena occurring during a thermal scan of nanofillers and PN, such as melting, crystallization, cure kinetics, and glass transition. These properties present a peculiar change when dispersion at nanoscale is achieved. Dynamic mechanical thermal analysis (DMTA) was frequently used in nanocomposites characterization since it allows the measurement of stiffness and energy losses as a function of temperature. DMTA data are strongly affected by the degree and the scale of dispersion of nanofillers. Thermal mechanical analysis (TMA) was mainly used to measure the coefficient of thermal expansion (CTE) of nanocomposite materials in comparison with those of the matrix. Thermogravimetric analysis (TGA) has been used to analyze the effect of the introduction of nanofillers into a polymer matrix on the thermal stability of the polymer. The literature concerning the analysis of the thermal stability and degradation of PN by TGA covers numerous original papers as well as comprehensive review articles. Polymer decomposition, either in presence of oxidative or non oxidative gas, significantly depends on the presence of fillers and on their dispersion scale.

Most reviews deal with the effects of different nanofillers on the properties of polymer matrices. In particular, Vyazovkin has published detailed review articles on thermal analysis literature. In these reviews, as well as in much of his own work, he provides considerable insight into use of TA methods for investigating the properties of polymer nanocomposites, including limitations [28,29,30].

However, this work is not intended to be a comprehensive review on the thermal properties of polymeric nanocomposites, but rather to illustrate the versatile applications of thermal analysis (TA) in the emerging field of polymer nanomaterial research. Thermal analysis, in fact, through analyzing bulk samples of several milligrams sizes, is capable of providing information on the average structure of the nanocomposite, even at the nanoscale. Therefore, this review will address the most relevant results obtained by TA that can be used to obtain indirect evidence of the nanodispersion, highlighting the strong potential of such instruments, as low cost techniques frequently available in most industrial and research laboratories. Wilkie has also published extensively on this topic and has demonstrated that synthetic anionic clays, such as layered double hydroxides (LDHs), do not exhibit a correlation between dispersion and montmorillonite nanocomposites [31,32].

## 2. Results and Discussion

### 2.1. Differential Scanning Calorimetry (DSC)

Differential scanning calorimetry has been widely applied in the investigation of numerous phenomena occurring during the thermal heating of organoclays and polymer/clay nanocomposites or nanotubes, involving glass transition (T_g_), melting, crystallization and curing.

The DSC method is one of the most common techniques applied to investigate the α-transition in polymers and their composites [33]. The α-transition is related to the Brownian motion of the main chains at the transition from the glassy to the rubbery state and the relaxation of dipoles associated with it.

Referring to clay nanocomposites, DSC technique is able to highlight appreciable enhancements in T_g_ brought about by the presence of nanosized montmorillonite in many polymers. This effect was typically ascribed to the confinement of intercalated polymers within the silicate galleries that prevents the segmental motions of the polymer chains. In the case of polyurethane (PU)—urea nanocomposites, the changes in the glass transition temperature were also interpreted as a result of effective links between polymeric chains and the silicate surface [34]. It was pointed out that these anchored polymer chains could form an interphase region, where the segment relaxation was slower than in the bulk. The restricted relaxation behavior for the polymer nanocomposites with intercalated and exfoliated silicates primarily depended on the exfoliation extent of the layered silicates and on the interaction strength between the silicate surfaces and the PU macromolecules.

**Figure 1:**
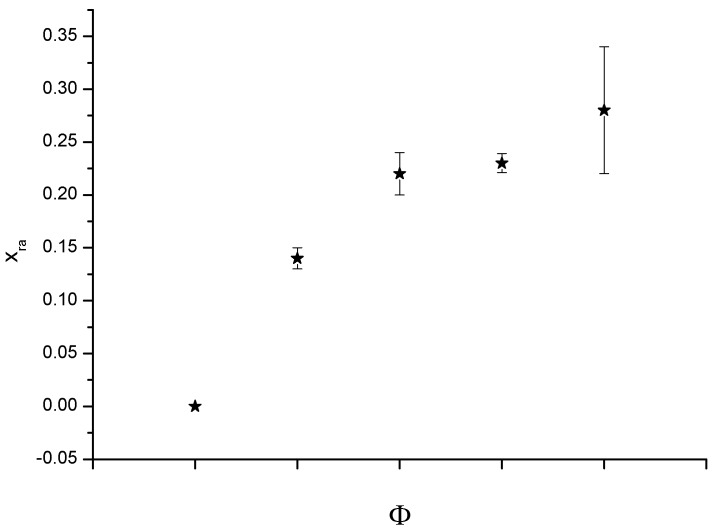
Rigid amorphous fraction of the polyurethane (PU) nanocomposites.

Nanocomposite adhesives obtained using a montmorillonite, modified with organic cations (OMMT), in a polyurethane matrix were recently synthesized and characterized by Esposito Corcione *et al*. [35]. A mix of exfoliated and intercalated layers was obtained, as ascertained by the measurements of structural and macroscopic properties of the nanocomposites. A significant increase of T_g_ of the nanocomposites as function of OMMT content was also observed by calorimetric analysis, confirming the mentioned limitations to chain segment mobility induced by OMMT. The intercalation of polyester polyol between OMMT lamellae was easily achieved by mixing. The presence of a significant fraction of rigid amorphous phase (x_ra_) was also revealed by calorimetric analysis. The rigid amorphous part of the polymer is defined by Esposito Corcione *et al*. [36] as being non crystalline (as measured by heat of fusion), but possessing above the glass transition temperature, Tg (of the “mobile amorphous” part) still a Cp (heat capacity) indicative or the solid state (vibrational motion only, continuous to the glassy Cp below Tg). The rigid amorphous fraction of the PU nanocomposites increased with increasing volume fraction (Φ) of OMMT as reported in Figure 1.

The segmental mobility was significantly reduced as OMMT content increased, indicating that PU chain immobilization occurs when they are intercalated between OMMT lamellae. In analogy with the interpretation of rigid amorphous fraction used for semi crystalline polymers, the Heterogeneous Stack Model was considered closer to the behavior of these nanocomposites. It describes the organization of the lamellae of a semi-crystalline polymer within the spherulites. HET makes the assumption that the entire mobile amorphous region (MAF) is outside the lamella stacks and the rigid amorphous fraction is the only amorphous material located between adjacent lamellae. Furthermore, a substantial constant value of the characteristic size of the cooperativity rearranging region (ζ*_a_*) at T_g_ was calculated. The existence of cooperativity rearranging regions is a basic concept in several theories and models of glass transition. Basically it means that the rearranging movement of one particle is only possible if a certain number of neighbor particles are also moved. The molecule do not relax independently of one another and that the motion of a particular molecule depends to some degree on that of its neighbors.

In a different work, the data obtained by Temperature Modulated Differential Scanning Calorimetry (TMDSC) showed the relationship between interlayer distance (Δ*d*) and the increment of heat capacity (Δ*C*_p_) for PU/clay intercalated nanocomposites [37]. The Δ*C*p values of nanocomposites with interlayer distances smaller than the characteristic length ζ*_a_* of bulk polyurethane (1.45 nm), were reduced. However, for nanocomposites with interlayer spacing larger than 2 nm, cooperative rearranging of polyurethane was substantially unmodified by the presence of the nanofiller, and Δ*C*_p_ values remained the same as that of bulk polyurethane.

**Figure 2:**
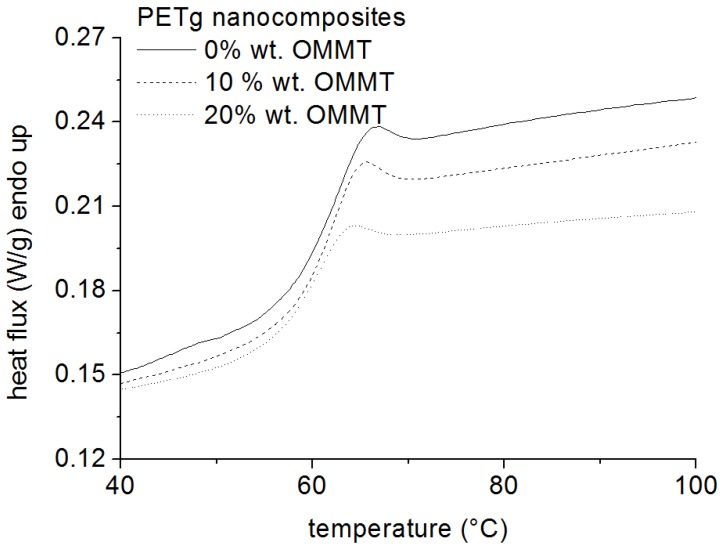
Glass transition temperature of nanocomposites based on amorphous poly(ethylene terephthalate) (PETg) and organically modified montmorillonites.

Other nanocomposites, however, showed a lower glass transition temperature, or were characterized by no change in the T_g_, compared to the neat polymer [38]. Glass transition temperature of nanocomposites based on amorphous poly(ethylene terephthalate) (PETg) and organically modified montmorillonites, obtained by melt intercalation, was also found to slightly decrease by Greco *et al*. [39,40,41,42], as proved by using the DSC technique. The neat PETg was completely amorphous, with a T_g_ of about 68.1 °C. After the addition of the nanofiller, the T_g_ decreased to 67.1 °C and to 66.0 °C for the samples with 10%wt. and 20% wt. of OMMT respectively, as shown in Figure 2.

The absence of any other exothermal or endothermal signal confirmed that the nanocomposite was amorphous. This is in agreement with the results reported by Kattan *et al*. [43], who observed that the PETg keeps its amorphous state in most practical experimental conditions. These decreases of T_g_. can be attributed to the plasticization effect of the organic modifier of the OMMT, characterized by a very low T_g_. The authors also suggest that the decrease in Tg could not be attributed to the organic modifier. If this is the case, all clay nanocomposites should show a lower Tg. Again, this is likely a more general effect of the specific enthalpic interactions. The effect of the nanoparticle on the Tg can be explained by the enthalpic interactions between the polymer and the nanoparticles. Either an increase or decrease in Tg can be induced depending on the specific interactions, as reported by Lewis [44].

The work by Torre *et al*. showed the strong influence of the preparation route on the thermal properties of polystyrene (PS) nanocomposites [45]. An appreciable reduction in the T_g_ was observed only for composites obtained from solution, whereas the composites obtained by melt intercalation showed T_g_ values approximately equal to that of neat polymer. The decrease in Tg could also be attributed to trapped solvent. Some difficulties in detecting changes in T_g_ for polymer-clay nanocomposites occurring with conventional DSC [46] could be overcome using TMDSC method.

DSC analysis was also used to measure the T_g_ of CNT- nanocomposites, such as in the case of coiled carbon nanotubes (CCNT) dispersed in an epoxy matrix [47]. Compared to the neat epoxy, a shift of T_g_ to higher temperatures was observed in the composites with single-walled carbon nanotubes (SWNTs) and multi-walled carbon nanotubes (MWNTs), while there was a decrease in T_g_ in the CCNT/epoxy composites. The ΔH of polymerization of the SWNT/epoxy composites was higher than that of the unfilled epoxy, while the ΔH calculated for the MWNT/epoxy and CCNT/epoxy composites were both lower than that of the neat epoxy. In particular, the ΔH of MWNT was slightly lower than that of neat resin, whereas for CCNT the ΔH was significantly lower than that of the neat epoxy. It was inferred that during the glass transition process, SWNTs act as a heat sink to accelerate the heat absorption of the epoxy, while CCNTs act as heat-shielding filler and prevents the epoxy from exchanging energy. In epoxy nanocomposites epoxy it is usually adsorbed on the nanoparticle due to lower surface energy as compared to the curing agent. This phenomenon causes segregation and variation of the Tg at the interphase [48]. The observed changes revealed that the tube surface configuration plays an important role in the glass transition behavior of epoxy matrices. It has been demonstrated that the incorporation of carbon fillers can affect the structure of the cured epoxy by restricting the nucleophile–electrophile interaction during the cure reaction by a steric hindrance effect. Accordingly, nanotubes with different shapes would have different steric hindrance effects on the cure reaction occurring in the epoxy in presence of the hardener. The particular helical shape of CCNTs should have a more steric hindrance effect than the straight nanotubes, such as SWNTs and MWNTs. Steric hindrance increases with the ratio surface-to-volume of a nanoparticle. This is not the case here, so steric hindrance is not the reason. The surface of carbon based nanoparticles can be different due to impurities, OH, COOH groups, metallic vs. semimetallic nanotubes, *etc.* All these factors affect the nanoparticle-polymer interactions. As a result, the cure reaction of the epoxy would be influenced more by CCNTs than by the SWNTs and MWNTs.

**Figure 3:**
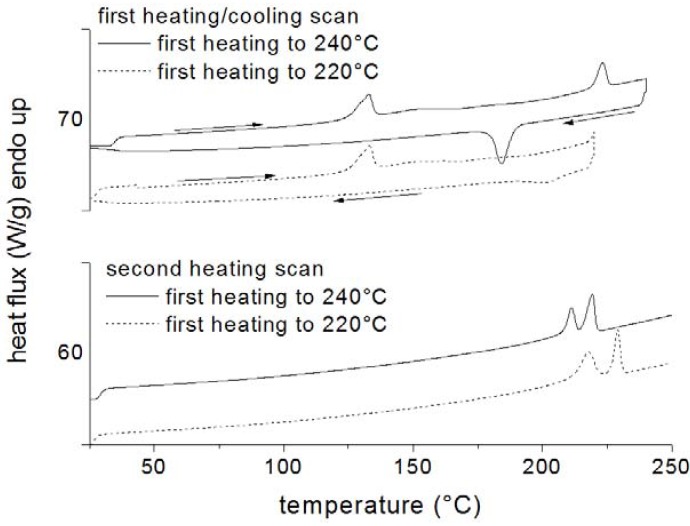
Differential scanning calorimetry (DSC) curves of the CBT01(unfilled) and CBT13MMT(filled with organically modified montmorillonite (OMMT)) systems.

**Figure 4:**
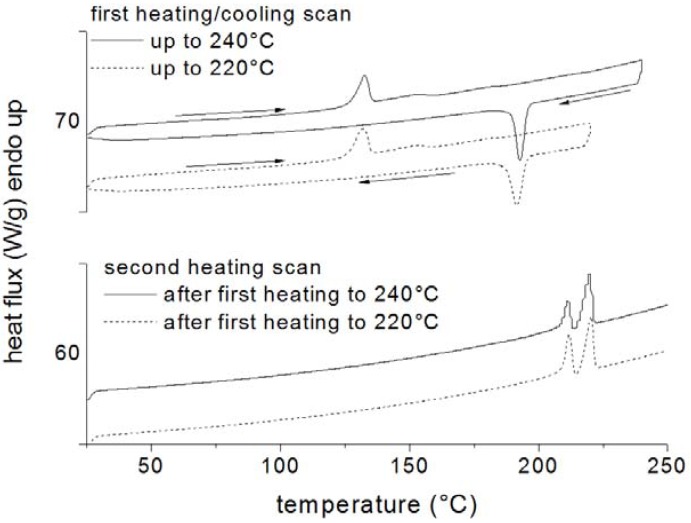
DSC curves of the CBT01 (unfilled) and CBT13MMT (filled with OMMT) systems.

Differential scanning calorimetry was also used by Greco *et al*. [49] to study the thermal characteristics of the species produced during the ring opening polymerization of cyclic butylene-terephthalate (CBT). The effects of the addition of small amounts of sodium and organo-modified montmorillonite on a tin-catalyzed polymerization were analyzed. It was found that the addition of the nanofiller significantly affected the polymerization of CBT, shifting the onset of the polymerization reactions to higher temperatures. This is evident by comparing DSC curves of the CBT01(unfilled) and CBT13MMT (filled with OMMT) systems heated up to different temperatures in an first heating scan, reported in Figure 3 and Figure 4 respectively.

This behavior was attributed to the tin catalyst being adsorbed in the lamellar galleries of the filler, thus reducing its activity for the polymerization reactions. As a consequence, CBT was formed at higher temperatures, preventing it from crystallizing during the heating scan. However, the nanofiller acts as efficient nucleating agent for the crystallization during the cooling cycle. When long isothermal holding times are used at temperatures lower than the equilibrium melting point of poly-butylene-terephthalate (PBT), the resulting polymer can crystallize to some extent and the crystal so produced has a nucleating effect on the crystallization taking place during the subsequent cooling cycle.

**Figure 5:**
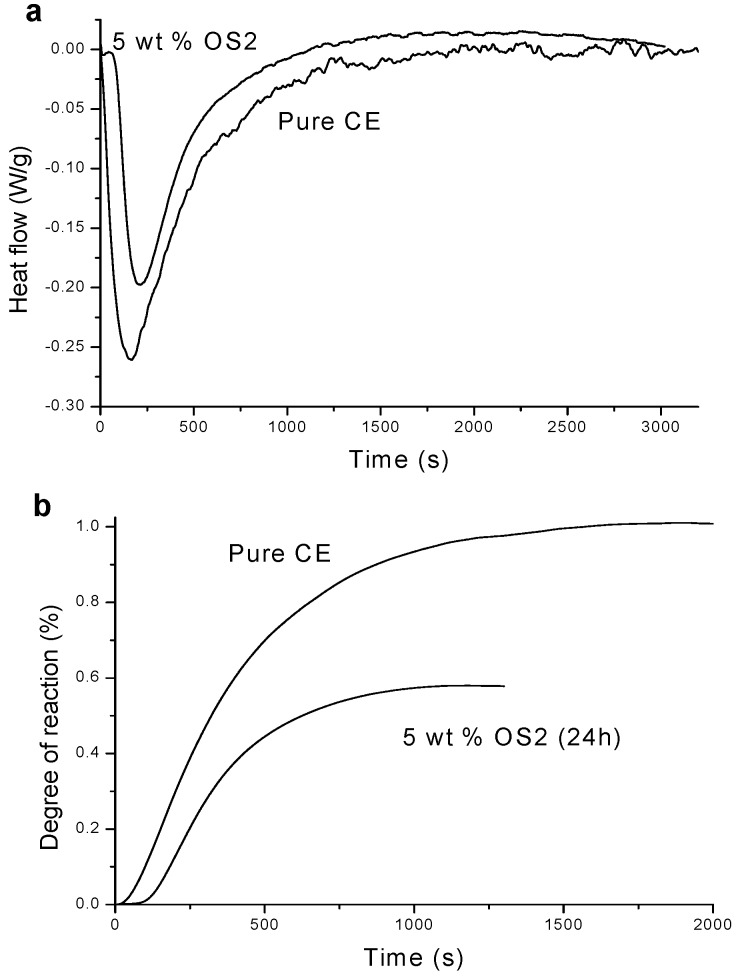
Kinetic behavior of novel nanocomposite coatings based on a cycloaliphatic epoxy resin (CE) with two different o-Boehmites (OS1 and OS2).

The DSC study of the curing process and its kinetics can give insight into the actual mechanism of cure reaction and its effect on degree of cross-linking and, as a consequence, on mechanical properties. A proper DSC, modified for irradiation of the sample using a UV light source (p-DSC), was used by Esposito Corcione *et al*. [50] to study the kinetic behavior of novel nanocomposite coatings based on a cycloaliphatic epoxy resin (CE) with two different o-Boehmites (OS1 and OS2), prepared by photochemically initiated cationic polymerization. The reactivity of the nanocomposites matrix was found lower than that of the neat resin, as shown in Figure 5(a,b) in the case of the nanofiller OS2. This behavior was attributed to the light shielding of the Boehmites as a consequence of scattering due to the presence of clusters in the micron-size range.

On the other hand, the results of a DSC analysis performed to investigate the curing process of nitrile rubber (NBR)/layered clay nanocomposites showed a reduction in curing time and an increase in enthalpy of curing in the systems with organoclay; autocatalytic model showed the best fit in kinetic modeling [51]. However, a little change in the cure behavior of the NBR/unmodified clay system, if compared to (NBR)/layered clay nanocomposites, was observed, suggesting that the accelerating effect was due to the introduction of ammonium modifier to OMMT. Furthermore, the cure kinetics studies on natural rubber–organoclay [52] and fluoroelastomer/organoclay [53] nanocomposites showed the suitability of the autocatalytic model for analyzing the cure parameters of rubber/clay nanocomposites. DSC analysis of cure kinetics of fluoroelastomer nanocomposites confirmed the catalytic role of organic modifiers on vulcanization process, whereas the opposite effect, consisting in slowing down the curing reaction, was observed in systems based on unmodified clay. Moreover, in DSC experiments a catalytic effect of ammonium compounds on homopolymerization of epoxy resin was observed [54,55]. In Seo *et al*. work, an increase in curing rate with increasing clay content was considered to be due to the presence of OH groups of the organic modifier of the clay, which could accelerate the epoxy curing reactions [56].

In a paper by Tian and Tagaya, DSC technique was used to investigate the influence of preparation route for nanocomposite materials based on poly(lactic acid) on the polymer morphology. It was found, in fact, that the melting enthalpy of poly(lactic acid)/montmorillonite (PLA/MMT) nanocomposites changed with an increase of nanoadditive content, depending on the method of nanocomposite preparation [57]. The change in melting enthalpy, ΔH, of the PLA/MMT system prepared by melt extrusion was considerably higher than that measured on systems produced by solvent dissolution method. It was suggested that the binding force between PLA and the inorganic compound in the composite prepared by melt extrusion method was higher than that relative to the system prepared by solvent dissolution method.

The value of melting enthalpy, measured by the DSC method, is commonly used to calculate the degree of crystallinity. In poly(ethylene oxide) (PEO)/organically modified montmorillonite (OMM) nanocomposites the degree of crystallinity continuously decreased with increasing the clay content [58]. A decrease in the degree of crystallinity has also been reported to occur, in polyethylene [59], low density polyethylene (LDPE) [60], poly(vinylidenefluoride) [61] and polyamide-6 [62] nanocomposites reinforced with OMM or with bentonite clays.

Kinetic analysis of the polymer crystallization by DSC in isothermal conditions can provide information about the effect of nanoparticles on the mechanism of nucleation and crystals growth. The reduction of the half-crystallization time (*t*_1/2_) was considered an evidence of the crystallization rate of polypropylene at low OMMT contents [63]. *t*1/2 was lower than that of neat polymers for the crystallization process of many polymer matrices modified with OMMT [64,65].

The kinetic analysis of isothermal crystallization of polypropylene-grafted with maleic anhydride copolymer (PP-*g*-HMA) based nanocomposites showed significant changes of the Avrami exponent *n*, suggesting the change of the crystal growth process from a three-dimensional crystal growth characteristic of the pristine polymer to a two-dimensional spherulitic growth for the nanocomposites.

In another paper, the Avrami plots showed that the crystal growth of PE in the intercalated sample is two-dimensional, while it is three-dimensional in the exfoliated sample. The activation energy for the crystallization of the intercalated sample is slightly lower than that of the exfoliated sample [66].

DSC was also applied to study the influence of nanoparticles on the polymer matrix morphology formation. As an example, the polymorphic behavior of PA-6 upon the addition of OMMT was studied, using this technique [67,68,69]. During DSC heating scan, neat PA-6 is likely to show only one endothermic peak at a temperature around 225 °C, which was associated with the melting of α-form crystals (T_m_,α) [70]. When the same polymer was modified with OMMT, an additional endothermic peak was observed at about 215 °C, corresponding to the temperature of melting of the less stable γ-form crystals (T_m,γ_). OMMT was in fact able to enhance the formation of γ-form crystals in PA-6 matrix, especially when crystallization took place in a lower temperature range. The origin of the new peak was explained in terms of the melting of a specific fraction of lamellae formed under stress in the volume of intercalated nanoclay sheets and tethered on the host layers by strong (interfacial) ionic interaction. The calorimetric results indicated also that the hybrids with small amounts of clay presented lower activation energy than PA-6 matrix, whereas those with higher clay loadings showed greater activation energy than PA-6 matrix.

Differential scanning calorimeters have been finally used to measure the thermal conductivity of Graphite Intercalated Compounds (GICs), together with other techniques [71]. The DSC method gave good results for thermal conductivities lower than 1.5 W m^–1^ K^–1^ but diverged from other more reliable techniques at higher values, which suggests that the DSC is suitable to measure conductivity for materials having thermal conductivities less than about 1 W m^–1^ K^–1^.

### 2.2. Thermogravimeric Analysis (TGA)

Thermogravimetric analysis performed on numerous PN showed that many polymers filled with montmorillonite and carbon nanotubes exhibited improved thermal stability (*i.e.*, a higher temperature for the onset of thermal degradation), such as in the case of poly(methyl methacrylate) (PMMA) [72], poly(dimethylsiloxane) (PDMS) [73], polyamide (PA) [74,75] and polypropylene (PP) systems [76].

It is usually well accepted that in the case of polymer–clay nanocomposites the improved thermal stability of the PN is mainly due to the formation of a char which hinders the out-diffusion of the volatile decomposition products, as a direct result of the decrease in permeability, usually observed in exfoliated nanocomposites [77]. Despite this, the exact degradation mechanism is currently not clear; such behavior is probably associated with the morphological changes in proportion relative to exfoliated and intercalated species with the clay loading. At low clay loading (*ca*. 1 wt.%), exfoliation dominates but the amount of exfoliated nanoclay is not enough to enhance the thermal stability through residue formation [78]. In addition, in air atmosphere, clay may slow down oxygen diffusion and thus produce thermo-oxidative reactions. On the other hand, the effect of clay on thermal stability in nitrogen is system dependent; therefore, there is no scientific evidence that there is an increase in thermal stability due to a decrease in permeability. When increasing the clay concentration (2 wt %–4 wt %), much more exfoliated clay is formed, char forms more easily and effectively and, consequently, promotes the thermal stability of the nanocomposites. At even higher clay loading level (up to 10 wt %), the intercalated structure is the dominant population and, even if char is formed in high quantity, the different morphology of the nanocomposite probably does not allow the maintaining of a high thermal stability. However, it is known that the chemical nature of the polymers, the type of clays and their modification route play an important role in the degradation behavior of PN. TGA measurement could also give indirect information about the amount of exfoliated nanoclay in the PN. However, char formation could not affect thermal stability since it is obtained at the very end of the decomposition.

Two important works review the thermal properties and degradation processes of nanocomposites based on different polymer matrices [77,79]. They discuss the basic changes in thermal behavior of different polymeric matrices (polyolefins, polyamides (PA), styrene containing polymers, poly(methyl methacrylate) (PMMA), poly(vinyl chloride) (PVC), polyesters, polyimides (PI), epoxy resins, polyurethanes (PU), ethylene–propylene–diene terpolymer (EPDM), poly(vinyl alcohol) (PVA), and polylactide (PLA) upon addition of montmorillonite, with special focus on the influence of montmorillonite on the kinetics of the degradation process and the formation of condensed/volatile products in oxidative and pyrolytic conditions. The results of recent research reported in the mentioned reviews [77,80] indicate that the introduction of layered silicates into polymer matrix causes an increase in thermal stability. Due to the characteristic structure of layers in a polymer matrix and their shape and dimensions close to molecular level, several effects have been observed that can explain the changes in thermal properties. Experimental results have shown that layers of MMT are impermeable to gases, meaning that both intercalated and exfoliated structure can create a labyrinth for gas penetrating the polymer bulk. Thus, the effect of a “labyrinth” limits the oxygen diffusion inside the nanocomposite during thermal degradation. Similarly, in the samples exposed to a high temperature, the MMT layers restrain the diffusion of gasses evolved during degradation, contributing to keep the neat polymer in contact with a non-oxidizing environment. Moreover, MMT layers are thought to reduce heat conduction. In the presence of MMT layers, strongly interacting with polymer matrix, the motions of polymer chains are limited, as explained in the former section dealing with Tg changes induced by OMMT. This effect brings additional stabilization in the case of polymer/MMT nanocomposites. The heat barrier effect could also provide superheated conditions inside the polymer melt leading to extensive random scission of a polymer chain and the evolution of numerous chemical species which, trapped between clay layers, have more opportunity to undergo secondary reactions. As a result, some degradation pathways could be promoted leading to enhanced charring. It is also suggested that the effect of more effective char production during thermal decomposition of polymer-clay nanocomposites may be derived from a chemical interaction between the polymer matrix and the clay layer surface during thermal degradation. Some authors indicated that catalytic effect of nanodispersed clay is effective in promoting char-forming reactions. Nanodispersed MMT layers were also found to interact with polymer chains in a way that forces the arrangement of macrochains and restricts the thermal motions of polymer domains [79,80]. Generally, the thermal stability of polymeric nanocomposites containing MMT is related to the organoclay content and dispersion. The synthesis methods influence the thermal stability of polymer/MMT nanocomposites as long as they are governing the dispersion degree of clay layers. Currently, extensive research is devoted to the synthesis of novel thermally stable modifiers (including oligomeric compounds) that can ensure good compatibility and improve the nanocomposite thermal stability due to low migration characteristics [79,80].

Using thermogravimetric analysis (TGA), several groups have also reported improved thermal stability in nanotube/polymer composites compared to neat polymers [14,15,16]. Specifically, the onset decomposition temperature, *T*_onset_, and the temperature of maximum weight loss rate, T_peak_, are higher in the nanocomposites. For example, Ge *et al*. [80] found that 5 wt % MWNT addition caused a 24 °C shift in *T*_onset_ as compared to that of the neat PAN. A number of mechanisms have been suggested. Dispersed nanotubes might hinder the flux of degradation products and thereby delay the onset of degradation. Polymers near the nanotubes might degrade more slowly, which would shift *T*_peak_ to higher temperatures. Another possible mechanism attributes the improved thermal stability to the effect of higher thermal conductivity in the nanotube/polymer composites that facilitates heat dissipation within the composite [81]. The observed improvement in thermal stability hints that nanotubes could be efficient as fire-retardant additives in polymer matrices.

A significant enhancement in thermal stability has been also recorded for Poly-Ethylene-Vinyl-Acetate (EVA) filled with the MWNT when compared to unfilled EVA: the two degradation steps of the EVA matrix (first the de-acetylation then the volatilization of resulting unsaturated chains) are shifted to higher temperatures with a decrease of the volatilization rate of the acetic acid and the formation of a stable char, which is further stabilized through *π-π* electronic interactions with the nanotubes [82].The effect on flame retardancy is concentration dependent. High loadings increase peak of HRR and ignition time (measured by cone calorimeter).

### 2.3. Dynamic Mechanical Thermal Analysis (DMTA)

DMTA was frequently used in nanocomposites characterization since it allows the measurement of two different moduli of the nanocomposites, the storage modulus (*E*’) which is related to the ability of the material to return or store mechanical energy, and the loss modulus (*E*’’), which is related to the ability of the material to dissipate energy as a function of temperature. DMTA data generally showed significant improvements in the storage modulus over a wide temperature range for a large number of polymer nanocomposites with MMT, such as PVDF [83], PP [84] and PMMA [85].

In nanocomposites based on PA-6 a linear increase in storage modulus with increasing the clay content was observed with a simultaneous decrease in intensity of the main relaxation peak [86]. The linear changes in modulus in a range of temperature below *T*_g_ was in accordance with coupling model, since in the glassy state both the amorphous and crystal phases of PA-6 have similar mechanical properties. Above the glass transition temperature, the amorphous phase become rubbery and the storage modulus changes from about 1 GPa to 1 MPa. The improvement of dynamic-mechanical properties of nanocomposites may be explained in terms of restricted thermal motions of polymer enveloped by clay nanoplates. The orientation of nanoparticles and higher-order structures of polymer influences the dynamic-mechanical properties as well. Apart from polymer morphology, the strength of interphase interactions was shown to be an important factor [87,88]. For this reason, DMTA technique could be used as an indirect method to have useful information about orientation, exfoliation and interphase interactions of nanoclay.

The introduction of a macromolecular compatibilizer, able to improve the interphase properties and facilitate the exfoliation of OMMT, was beneficial also in terms of dynamic-mechanical properties.

The DMTA analysis is also commonly used to determine the glass transition temperature of polymeric materials from the peak of loss angle tangent (tgδ) or from the maximum of loss modulus (E’’) [89]. However, it must be underlined that the glass transition value calculated using dynamic mechanical analysis is usually much higher (even 20 °C) than that measured by DSC, as clearly demonstrated in experimental papers [90,91].

**Figure 6:**
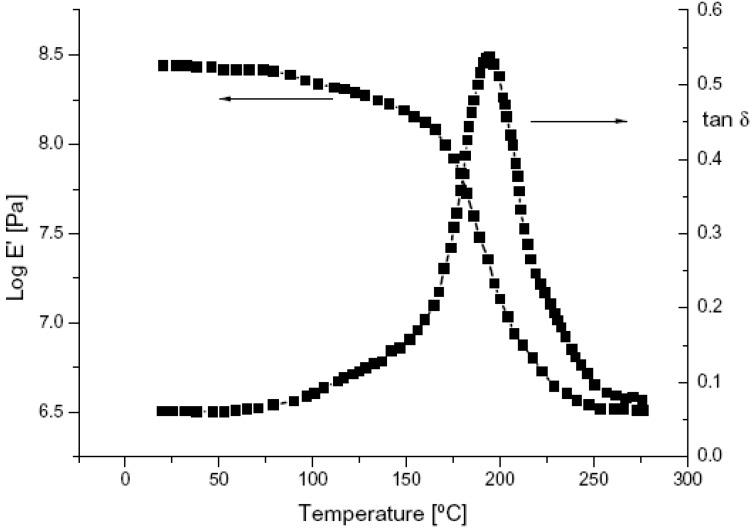
Typical dynamic mechanical thermal analysis (DMTA) spectrum of an UV-cured CE/OS1 (5 wt.%) nanocomposite.

The glass transition temperature (Tg) values of the pure UV-cured epoxy resin (CE) and of its o-boehmite(OS1) nanocomposites were measured by means of DMTA technique on samples previously exposed to a dynamic UV lamp, allowing a very high radiation intensity on the surface of the sample (1200 mW/cm^2^) [50]. A typical DMTA spectrum related to a UV-cured CE/OS1 (5 wt.%) sample is reported in Figure 6. The high Tg values obtained clearly indicate that the high intensity of the UV radiation can promote local overheating, determining a well advanced state of curing. A similar behavior was found in a previous work [92]. For all the products containing up to 5 wt.% of the nanofiller, the Tg was found very close to that of the pure UV-cured CE resin. By increasing the amount of the nanofiller in the UV-cured dispersion, a slight decrease of the Tg is evident, thus indicating a slight lowering of the crosslinking density of the polymer network. Since the slight decrease of Tg was observed also for the nanocomposite based on unmodified boehmite, this phenomenon cannot be only related to the presence of the sulfonic acids used for modifying the Boehmite nanoparticles in OS1 and OS2 (such modifying agents could in fact interfere with the photoinitiator in view of the high dissociation constant of the acid, which would produce large amounts of protons). Therefore, this behavior was mainly attributed to a scattering effect of the UV light due to the presence of the nanofillers in the polymer matrix [50].

A different trend was observed in the case of an epoxy matrix filled with the same boehmite nanoparticles (OS1 and OS2) and thermally cured [93]. Glass transition temperature, Tg, of the epoxy matrix nanocomposites was measured using dynamical mechanical analysis, in correspondence of the maximum of the loss modulus E’’. The loss modulus of nanocomposites with different concentration of boehmite (0%–15% wt.) was found to be significantly higher than that of the neat epoxy. An increase of the loss modulus in the glassy state about 50% was achieved as a result of incorporation of 10% wt. of the nanofiller into the epoxy resin. These losses are accompanied by an increase of Tg up to 9 °C for 10% of boehmite content. On the other hand E’’ was unaffected by the presence of the nanofiller in the rubbery state. Any further increase of nanoparticles concentration was associated to a lower increase of Tg, which can be detected using DMTA analysis. Another relevant feature of polymer nanocomposites is given by the decrease of the intensity and the broadening of the tan δ peak in correspondence of the glass transition temperature [94,95,96].

DMTA technique was also used to characterize carbon nanotubes nanocomposites [97,98]. As an example, Gou [98]. developed a new processing method for the fabrication of single-walled nanotube (SWNT)-reinforced nanocomposites: the nanocomposites were fabricated by infiltration of diluted epoxy resin through a bulky paper followed by hot pressing. The wetting of the nanocomposites was examined using scanning electron microscopy and atomic force microscopy. The results showed that the epoxy resin completely penetrated the bulky paper through the nanoporous structures. The thermomechanical behavior of the nanocomposites was assessed using DMTA by monitoring the storage modulus against temperature. The modulus of the neat epoxy resin was increased by the stiffening effect of the nanotubes. The weight fraction of the nanotubes in the nanocomposites induced a stiffening effect. A slight reduction in the Tg values of the nanocomposites containing nanotubes at various compositions was observed. Compared to the neat epoxy resin, there is no apparent Tg peak on the tan δ curves of the nanocomposites. Above Tg, the tan δ curve continuously increases rather than returning to the baseline. For neat epoxy resin, the chain segments cannot completely move below its glass transition. The very low value of tan δ for neat resin means that there is no significant energy loss. During the glass transition, the molecule segment absorbs enough energy and begins to move; however, the free space in the polymer is too small, so more energy is required for the resin molecules to move. At a temperature higher than Tg, a larger free volume is available in the polymer to allow the molecule segments to freely move, leading to a decrease in the tan δ curve. The nanotubes have a large surface area and they are at the same scale as the resin molecules in SWNT bulky paper-reinforced nanocomposites. Therefore, the resin chain segments strongly interact with the nanotubes with associated high. These strong interactions between the nanotubes and chain segments could be responsible for the high values of tan δ above Tg observed in CNT nanocomposites.

### 2.4. Thermal Mechanical Analysis (TMA)

TMA is a highly sensitive technique for the measurement of expansion and contraction of cross-linked or filled materials, including nanocomposites [99]. TMA was used to measure the coefficient of thermal expansion (CTE) of nanocomposite materials based on PA-6 [100,101], PP [102], PA [103], PS [104]. It has been generally found that CTE is lower in nanomaterials in comparison to unmodified polymer, especially for low contents of OMMT. In general, the extent of CTE reduction depends on the particle rigidity and on the dispersion of the clay platelets in the matrix and also on an efficient stress transfer to clay layers. It is believed that the retardation of chain segmental movement through incorporation of organically modified clays also leads to decrease in the CTE [105].

As an example, in order to investigate the anisotropy of thermal expansion of PA-6/montmorillonite nanocomposites, TMA measurements were performed on injection molded samples in the three orthogonal directions, *i.e.*, flow direction (FD), transverse direction (TD) and normal direction (ND). PA-6 modified with OMMT was found to exhibit lower values of CTE than pure polymer in the direction parallel to the melt flow during injection molding [101], while increased values of CTE were measured in the direction normal to the melt flow. The latter result suggested a non-uniform orientation of exfoliated platelets about FD, since perfect alignment of disk-like platelets in an isotropic matrix must yield identical expansion coefficients for both FD and TD. Chains may have more orientation along FD than TD, thus leading to lower thermal expansion. Of course, disparity between the two directions may also reflect differences in the orientation of polymer crystallites. This trend may be explained by platelet orientation and anisotropy effects.

TMA results may indirectly provide information about the spatial orientation of MMT layers in nanocomposite materials.

A trend of CTE similar the latter results was obtained by TMA measurements performed on the multi-walled carbon nanotubes (MWNTs) infused through and between glass-fiber tows along the through-thickness direction [106]. Both pristine and functionalized MWNTs were used in fabricating multiscale glass-fiber-reinforced epoxy composites. The CTEs of the resin and the resin/fiber system were tested by TMA with a ramp rate of 5 °C min^−1^.

It was supposed that the CTE of MWNTs is negative. Thus the addition of MWNTs, especially well-dispersed and functionalized MWNTs, may reduce the CTEs of the nanocomposites [107]. The thermal expansion curves demonstrated that the CTE at temperatures above Tg was much higher than the corresponding CTE at temperatures below Tg. At temperatures below the glass transition temperature (Tg), the CTE values of CNT-reinforced epoxy/glass-fiber samples were lower than that of epoxy/glass-fiber sample with only 1 wt% of MWNTs, since the CTE of MWNTs was negative. Moreover, a lower CTE was obtained in functionalized MWNT/epoxy/glass-fiber nanocomposites at temperatures below the Tg, which suggested that smaller CNT bundles produced smaller CTE values below Tg.

TMA can be also used to measure the glass transition, in terms of change in the CTEs, as the polymer turns from glass to rubber state with a dramatic change in free molecular volume. Thus, Tg can be determined from the thermal expansion curve. The reduction of Tg, in the presence of the nanofiller, indicated that the crosslinking density of functionalized MWNT/epoxy/glass fiber nanocomposite is obviously decreased because of the interference of functionalized groups on the MWNTs. However, the Tg of MWNT/epoxy/glass-fiber nanocomposite was almost the same as that of pure epoxy, which suggested that the pristine MWNTs did not participate in the epoxy curing reaction and were not fully integrated into the epoxy crosslinked network [107].

## 3. Conclusions 

Polymer nanocomposites (PN), *i.e.*, polymer composites reinforced with inorganic fillers of dimensions in the nanometer range, have attracted a great interest of researchers, due to unexpected synergistic properties derived from the two components.

In PN, the efficiency of intercalation of the polymer in the lamellar galleries is usually measured by means of X-ray diffraction (XRD) and /or transmission electronic microscopy (TEM). Although wide angle XRD offers a convenient method to determine the interlayer spacing of the silicate layers in the intercalated nanocomposites, little can be said about the spatial distribution of the silicate layers or on structural non-homogeneities in nanocomposites. On the other hand, TEM is very time-intensive, and only gives qualitative information on the sample as a whole, due to the small investigable area.

On the other hand, thermal analysis (TA) is a useful tool to investigate a wide variety of properties of polymers and it can be also applied to PN in order to gain a further insight into their structure, in particular in the case of montmorillonite nanocomposites. This review presents some useful examples of applications of differential scanning calorimetry (DSC), thermogravimetric analysis (TGA), dynamic mechanical thermal analysis (DMTA) and thermal mechanical analysis (TMA) for the characterization of nanocomposite materials.
